# Clinical Applications of Mineral Trioxide Aggregate: Report of Four Cases

**DOI:** 10.5005/jp-journals-10005-1053

**Published:** 2010-04-15

**Authors:** Rahul Hegde, Prashant M Battepati

**Affiliations:** 1 Professor and Head, Department of Pedodontics and Preventive Dentistry, Bharati Vidyapeeth University Dental College, Kharghar, Navi Mumbai, Maharashtra, India; 2 Senior Lecturer, Department of Pedodontics and Preventive Dentistry, Bharati Vidyapeeth University Dental College, Kharghar, Navi Mumbai, Maharashtra, India

**Keywords:** Apexification, apexogenesis, pulpotomy, MTA.

## Abstract

The greatest threats to developing teeth are dental caries and traumatic injuries. The primary goal of all restorative treatment is to maintain pulp vitality so that normal root development or apexogenesis can occur. If pulpal exposure occurs, then a pulpotomy procedure aims to preserve pulp vitality to allow for normal root development. Historically, calcium hydroxide has been the material of choice for pulpotomy procedures. Recently, an alternative material called mineral trioxide aggregate (MTA) has demonstrated the ability to induce hard-tissue formation in pulpal tissue. This article describes the clinical and radiographic outcome of a series of cases involving the use of MTA in pulpotomy, apexogenesis and apexification procedures and root perforations repair.

## INTRODUCTION

The quest for an ideal material with predictable sealability, good biocompatibility and increased moisture sensitivity still exists. Lots of effort was put into develop a new material and the result was a totally versatile material. Its formulation has physical properties, setting requirements and characteristics necessary for an ideal medicament and repair material. Mineral Trioxide Aggregate is a new biocompatible material with numerous exciting clinical applications in endodontics. It has also been used on an experimental basis by pedodontist for several years with good success rate. Reported herewith are four case reports where in MTA was used as material of choice for apexification, apexogenesis, pulpotomy and repair of root perforation respectively. An attempt has also been made to review the current dental literature of mineral trioxide aggregate.^1^

## CASE REPORT 1: APEXIFICATION

A 13-year-old boy reported to the Department of Pedo-dontics and Preventive Dentistry with the chief complaint of pain in upper right front tooth region. On examination there was an Ellis and Davey class 3 fracture with 11, tenderness positive with history of trauma 1 year back. Radiograph revealed incomplete root formation (Fig. 1A). On the same day access opening, working length and biomechanical preparation was done and canal was dried with paper point and filled with calcium hydroxide for disinfection for 1 week (Fig. 1B). After a week, Ca(OH)_2 _was removed and then with the help of carrier MTA was placed into apical region (Fig. 1C). Material was then condensed into apical area and access opening was sealed with temporary filling material (Fig. 1D). Placement of the material was confirmed with a radiograph and after a week, tooth was obturated with gutta-percha (Fig. 1E). After 6 months follow-up radiograph revealed well sealed and healthy apical region (Fig. 1F).

**Fig. 1A:**
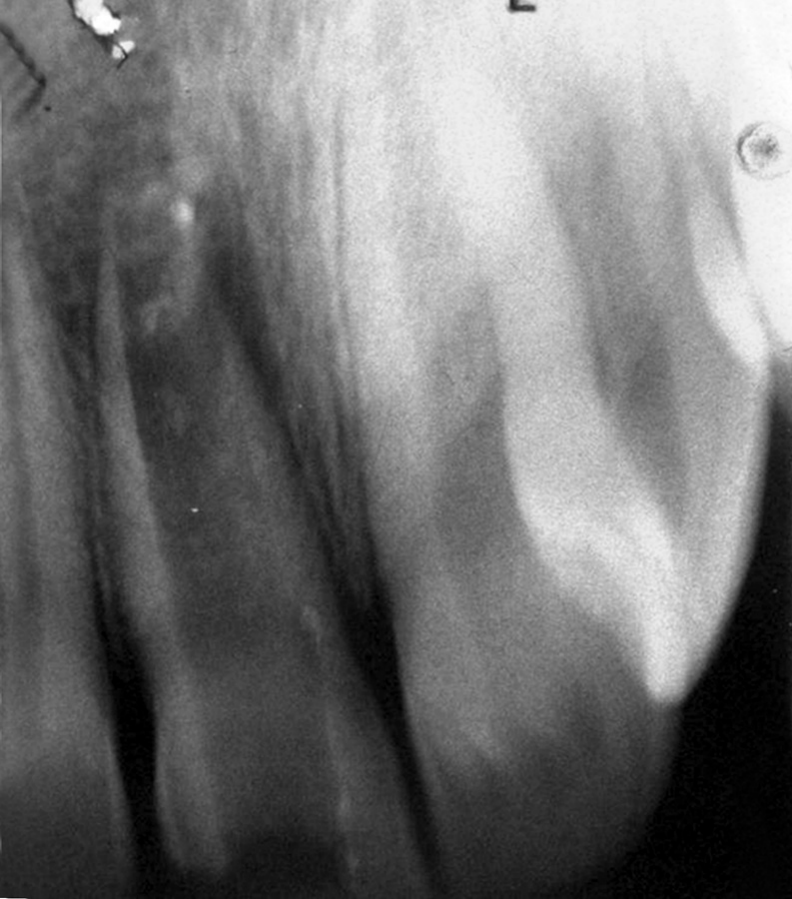
Preoperative radiograph of right central incisor

**Fig. 1B:**
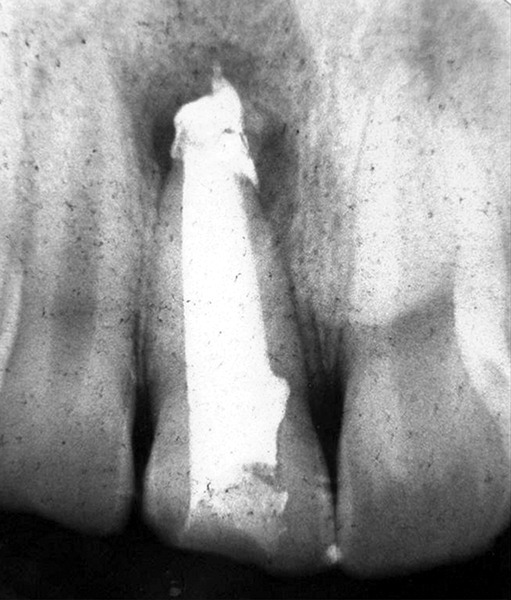
IOPA of calcium hydroxide placement in canal for disinfection

**Fig. 1C:**
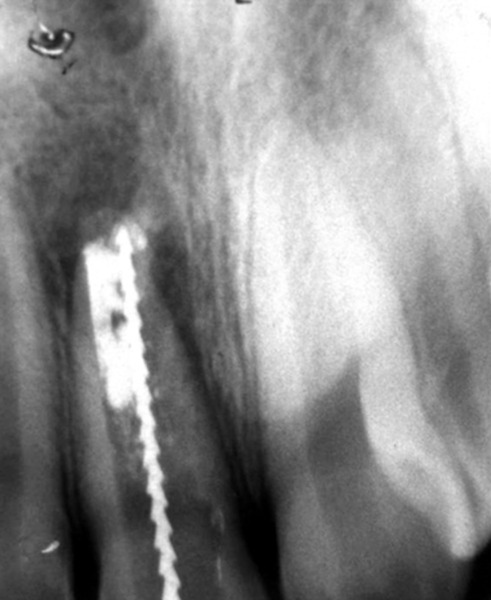
IOPA showing calcium hydroxide removed from canal after a week

**Fig. 1D:**
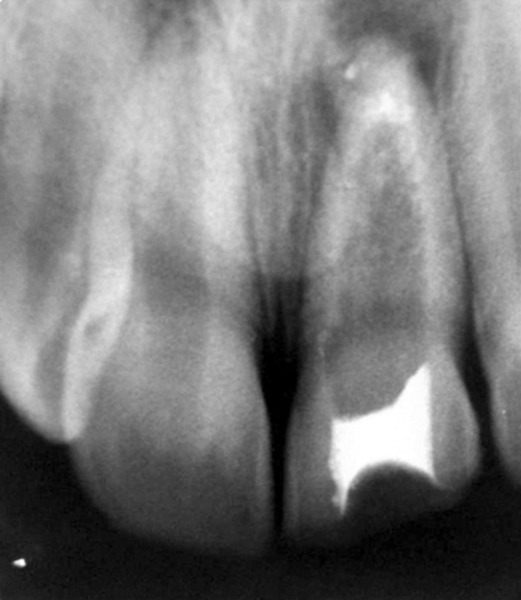
IOPA showing placement of mineral trioxide aggre gate at the apex and closure with temporary material

**Fig. 1E:**
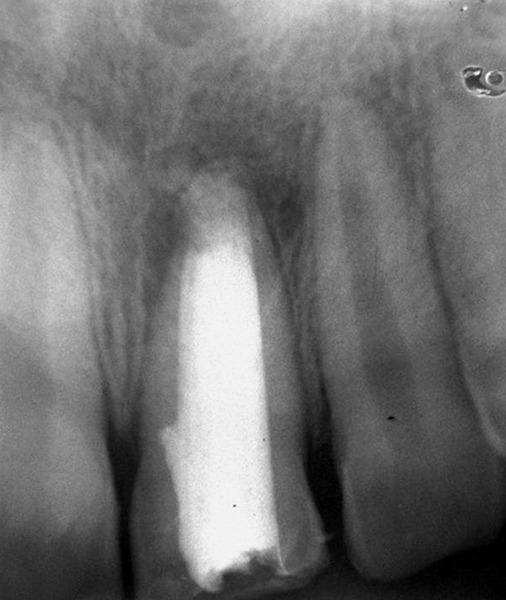
IOPA showing canal obturated with gutta-percha

**Fig. 1F:**
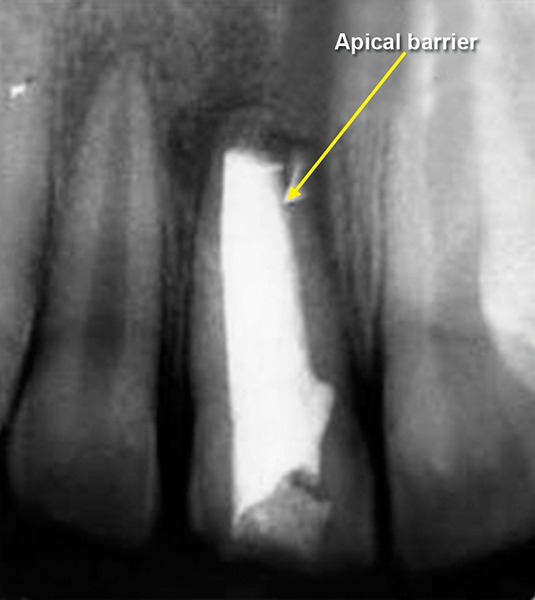
Intraoral periapical radiograph after 6 months showing apex fully sealed

## CASE REPORT 2: APEXOGENESIS

A 9-year-old girl reported to the Department of Pedodontics and Preventive Dentistry with the chief complaint of pain in upper right front tooth region (Fig. 2A). On examination there was a Ellis and Davey class 3 fracture with 11. Radiograph revealed incomplete root formation. Access opening was done followed by amputation of the coronal pulp with sharp excavator, carefully to prevent further damage to the pulp and perforation of the pulpal floor (Figs 2B and C). The coronal pulp tissue was removed, sterilized cotton pellets over each amputation site was once placed and pressure pack was applied for a few minutes. Once the homeostasis was achieved, 1 to 1.5 mm thick layer of freshly mixed mineral trioxide aggregate was placed directly on the pulp stump surface and then gently pressure applied with a moist cotton pellet. Damp cotton pellet was placed over the material, followed by temporary restoration with Zinc Oxide Eugenol Cement (Fig. 2D). After a week intermediate restorative material was removed and final restoration over set mineral trioxide aggregate was done followed by composite build up (Fig. 2E).

**Fig. 2A:**
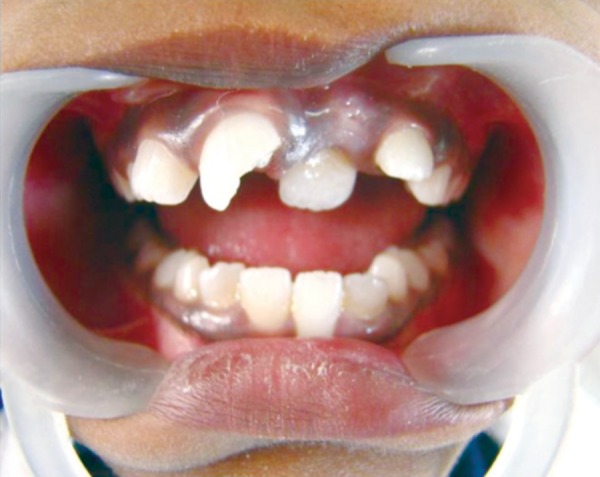
Intraoral view showing Ellis and Davey class 2 fracture with right central incisor

**Fig. 2B:**
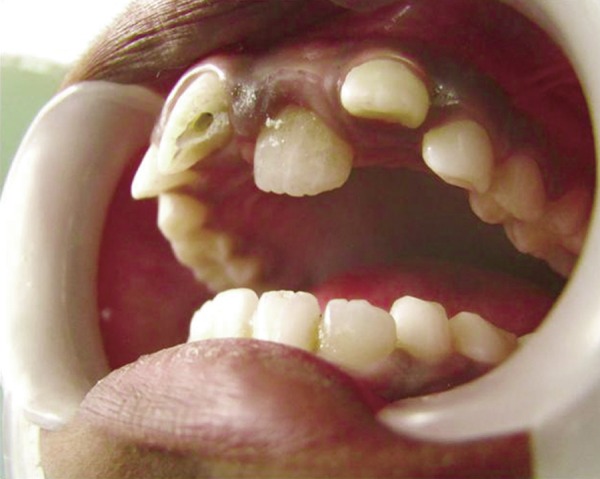
Intraoral view showing access opening with 11

**Fig. 2C:**
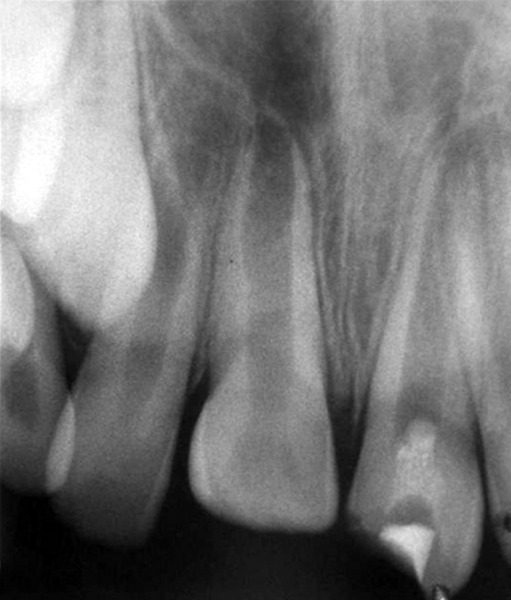
IOPA showing mineral trioxide placed in a coronal pulp

**Fig. 2D:**
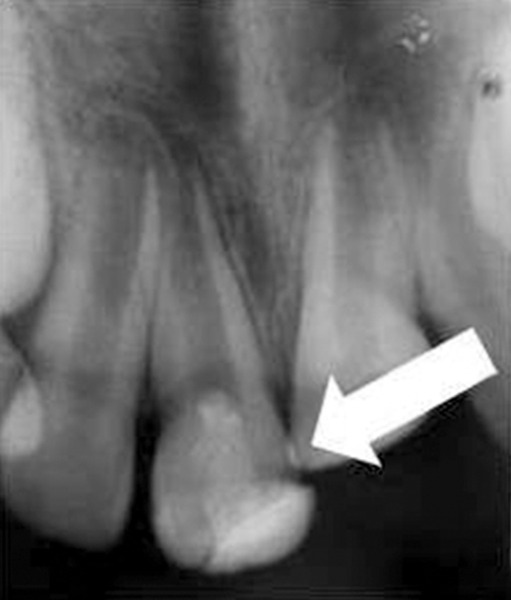
IOPA showing MTA in a pulp chamber and final restoration with composite

**Fig. 2E:**
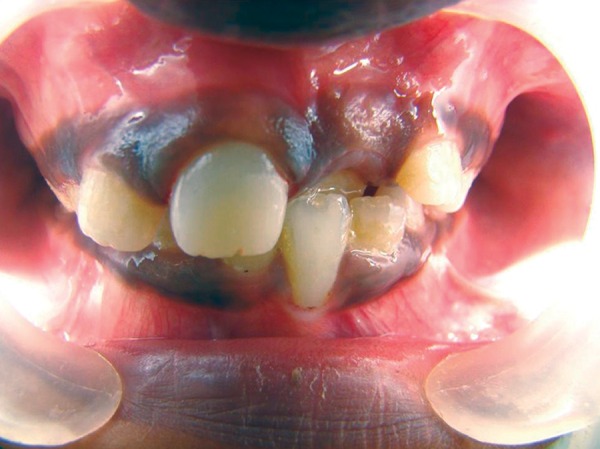
Intraoral view showing composite build up with 11

## CASE REPORT 3: PULPOTOMY

A 4-year-old boy came to Department of Pedodontics with chief complaint of carious lower back teeth. On examination, his all 4 mandibular primary molars were carious (Fig. 3A).

Radiograph revealed deep caries approaching pulp with remaining dentin thickness of about 0.5 mm (Fig. 3B). MTA pulpotomy was planned. Under rubber dam isolation all superficial caries was removed to minimize bacterial contamination before exposure. Then the roof of the pulp chamber was removed by joining the pulp horns with bur cuts followed by amputation of the coronal pulp with a sharp excavator (Fig. 3C). Following, coronal pulp amputation sterilized cotton pellets over each amputation site was placed and pressure was applied for 5 minutes. Then 1 to 1.5 mm thick layer of freshly mixed mineral trioxide aggregate was placed directly on the pulp stump surface and then patted with a moist cotton pellet (Fig. 3D). With a damp cotton pellet over the material, temporary restoration was done with zinc oxide eugenol cement (Fig. 3E). After a week intermediate restorative material was removed and mineral trioxide aggregate was placed, tooth was then restored with Glass Ionomer Cement (GIC) followed by stainless steel crown (Fig. 3F).

**Fig. 3A:**
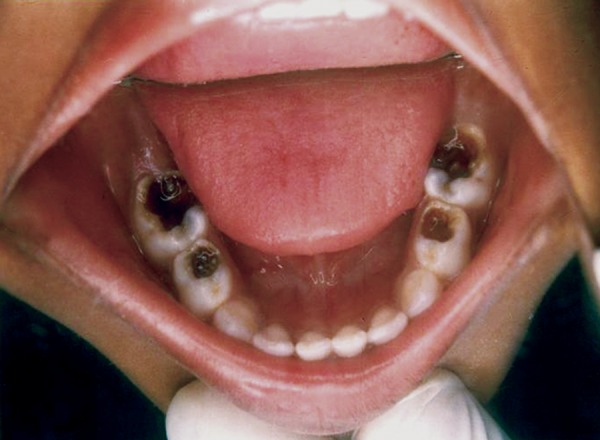
Intraoral view of lower carious molars

**Fig. 3B:**
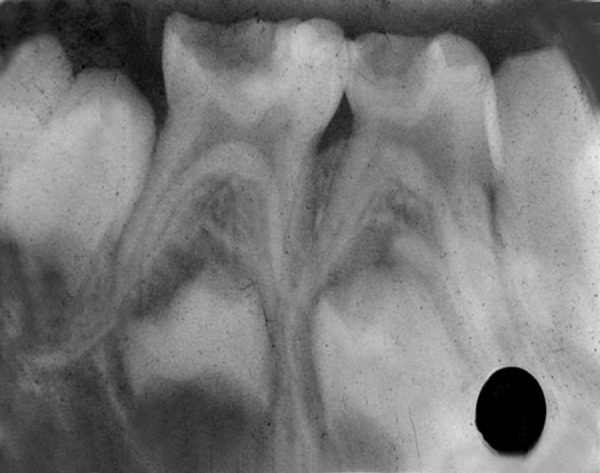
IOPA of 74,75 with remaining dentin thickness of 0.5 mm

**Fig. 3C:**
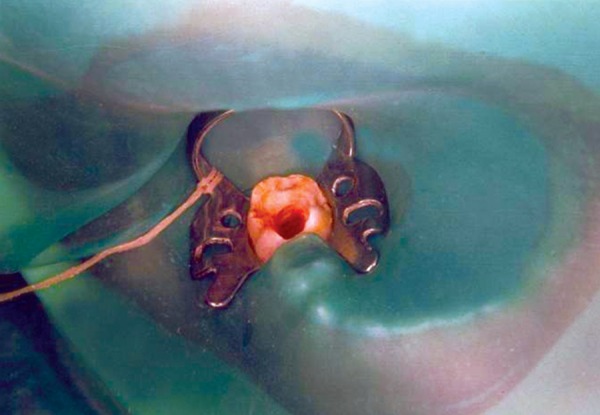
Intraoral view of lower molar with rubber dam isolation and access opening

**Fig. 3D:**
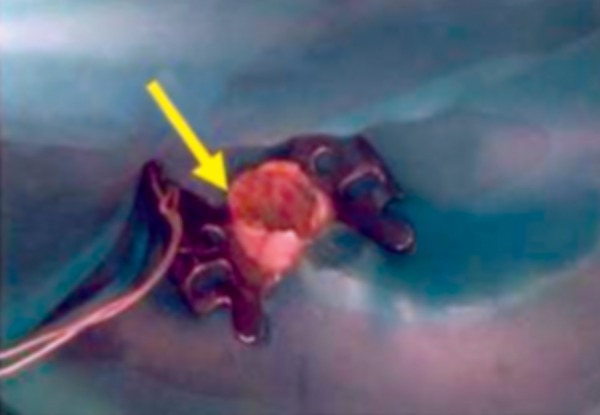
Intraoral view of lower molar with amputation of coronal pulp and MTA placement

**Fig. 3E:**
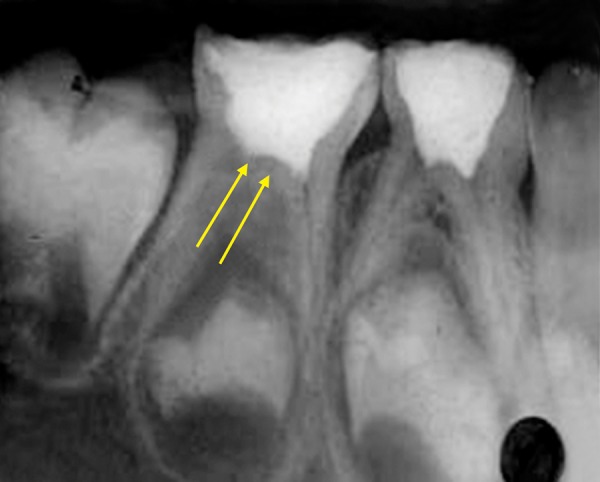
IOPA of pulpotomised 74,75

**Fig. 3F:**
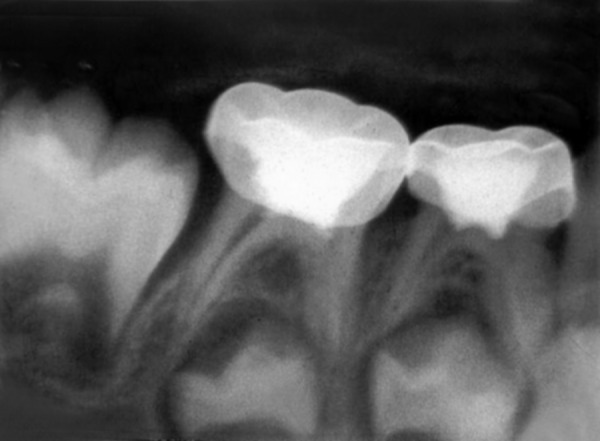
IOPA showing stainless steel crown on molars

## CASE REPORT 4: ROOT PERFORATION REPAIR

A 13-year-old girl came to Department of Pedodontics with chief complaint of fractured left central incisor. On examination, there was Ellis class 3 fracture in 21 with history of trauma 1 year back and treatment was done somewhere outside the college. The tooth had root canal and labial perforation on crown. Radiograph revealed incomplete root formation (Figs 4A and B).

The working length was measured and biomechanical preparation was done. Then canal was dried with paper point and filled with MTA on perforation site for repair and at the apex for apexification. Placement of mineral trioxide aggregate was confirmed with a radiograph for adequate barrier to be created, with a wet cotton pellet. Excess moisture was removed from the pellet and placed in the canal (Fig. 4C). Then temporary filling was removed at least after 3 to 4 hours and later a permanent filling material in the root and/or in the access cavity preparation was placed (Figs 4D and E). The healing was assessed periodically using intraoral periapical radiograph.

**Fig. 4A:**
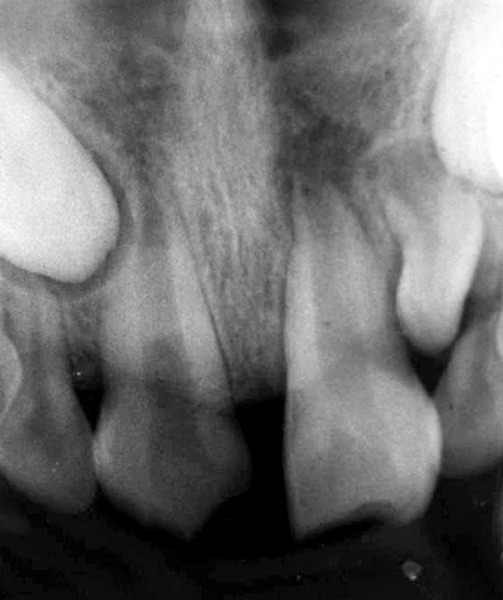
Preoperative radiograph of left central incisor with open apex and opened root canal

**Fig. 4B:**
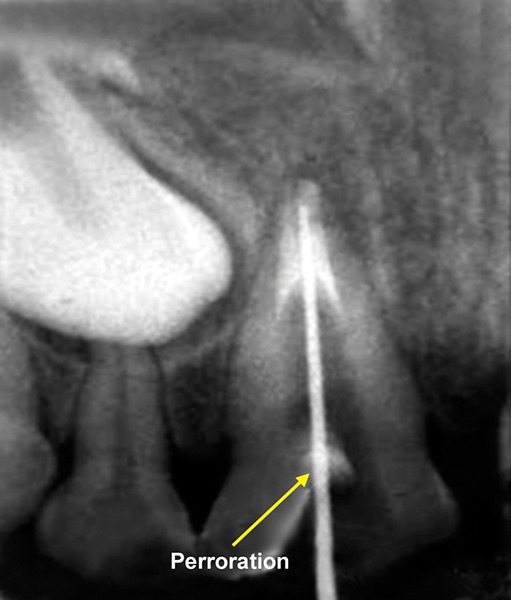
IOPA of MTA placed on labial perforation

**Fig. 4C:**
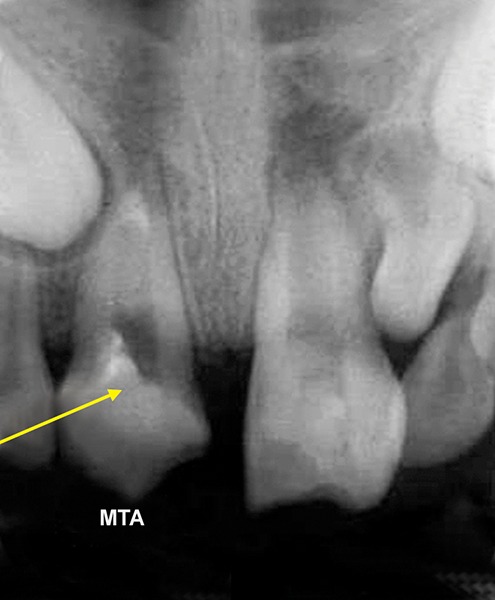
IOPA showing MTA on apex for apexification in 21

**Fig. 4D:**
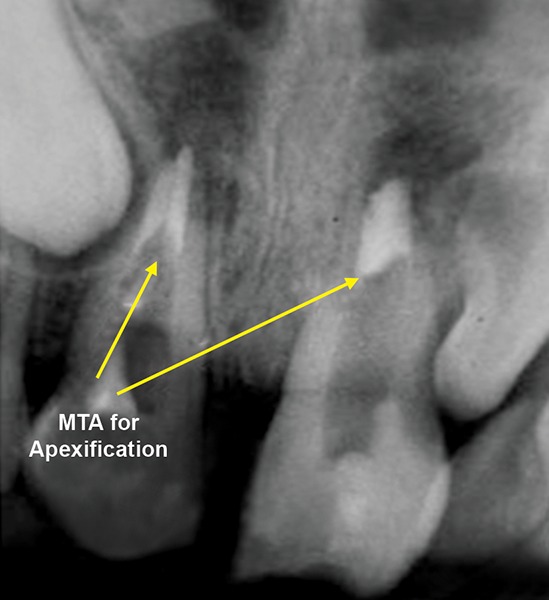
IOPA showing MTA on apex for apexification with 11, 21

**Fig. 4E:**
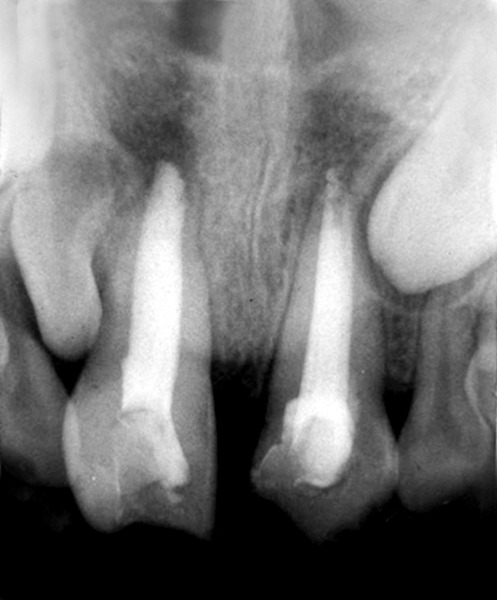
IOPA showing obturation with 11, 21

## PROPERTIES OF MTA

The pH of mineral trioxide aggregate after manipulation is 10.2 initially which rises to 12.^2^ 3 hours after mixing, and thereafter it remains constant and hydration of mineral triox-ide aggregate powder results in a colloidal gel that solidifies to a hard structure in less than 3 hours.^3,4^ In general terms the quicker the material sets the more it shrinks so compressive strength in the first 24 hours is 40 MPa which increases to 67 MPa after 21 days. MTA does not show any solubility. Radi-opacity is expressed as equivalent thickness of aluminium. Fhe radiopacity for mineral trioxide aggregate is 7.17 mm of equivalent thickness of aluminium.^5^ The biocompatibility of MTA can be investigated as-cytological investigations, subcutaneous and intraosseous implantation periradicular tissue reactions, Pulpal reactions. MTA has enhanced sealing ability which is attributed to its hydrophilic nature and setting expansion when it is used in moist oral environment. MTA has an antibacterial effect on some of the facultative bacteria but no effect on any anaerobic bacteria. The antibacterial effect of MTA against these organisms is because of its alkaline pH or release of diffusible substances into the growth medium which retards the growth of bacteria.^6^

## ADVANTAGES OF MTA OVER CALCIUM HYDROXIDE

Mineral trioxide aggregate takes less time for the management of the teeth with unformed apices than calcium hydroxide. It takes less time for the formation of biological barrier at the root apex than calcium hydroxide. It has low solubility and high compressive strength than calcium hydroxide. Calcium hydroxide offers no protection against microleakage, while MTA remains stable and resists micro-leakage after it sets. It stimulates cytokine release and inter-leukin production, which allows cementum overgrowth. So mineral trioxide aggregate produces less inflammation and better dentin bridge formation than calcium hydroxide.^6-9^

Mineral trioxide aggregate has demonstrated the ability to induce hard tissue formation in pulpal tissues and it promotes the rapid cell growth and it is believed that the deposition of hard tissue over the material is related to features like good sealing ability, biocompatibility and alkaline pH (Torabinejad, 1995), its capacity to attract blastic cells and to promote a favorable environment for cementum formation (Pittford,1995), presence of calcium and phosphorus ions in its formulation (Torabinejad, 1996), ability to stimulate cytokine release from bone cells and it also interact with the cell receptors, leading to change in synthesis of cellular RNA and protein and therefore in cell behavior.^8^

## DISCUSSION

MTA was developed by Dr Torabinejad at Loma Linda University in 1993. First described in the dental literature in 1993 for the repair of lateral root perforations. Mineral trioxide aggregate consists of 50 to 75% (wt), calcium oxide 15 to 25% (wt%) silicon oxide. When these raw materials are blended they produce-Tricalcium silicate, Dicalcium silicate, Tricalcium aluminate, Tetracalcium aluminoferrite. On addition of water the cement hydrates to form silicate hydrate gel. Radioopacifier-Bismuth oxide free crystalline silica which is in the form of insoluble residue. Trace elements- Calcium oxide (free Magnesium oxide, Potassium and Sodium sulfate compounds). MTA is available in two forms-grey mineral trioxide aggregate (GMTA), white mineral trioxide aggregate (WMTA). The major difference in the two is in the concentration of Carborundum (Al_2_O_3_), Periclase (MgO) and especially FeO, with values of each of these oxides being considerably lower in the white mineral trioxide aggregate than in grey mineral. The white mineral trioxide aggregate lacks the aluminoferrite phase that imparts the grey color to grey mineral trioxide aggregate.^1^ Recent studies showed that mineral trioxide aggregate stimulate cytokine release from bone indicating that it actively promotes hard tissue formation rather than being inert as any dental materials. MTA’s advantages are related to its ability to effectively seal the material tooth interface to prevent bacterial penetration and to its high level of biocompatibility. In contrast to calcium hydroxide, which deteriorates overtime and gradually disintegrates thereby leaving space for potential microleakage. MTA does not appear to change overtime. Therefore, it preserves the protective cover over, for instance developing reparative dentin, preventing bacterial invasion of the pulp. The pulp can tolerate almost any dental material and produce new dentin as long as it can be protected against microleakage, a function that MTA appear to perform better than any material with which it has been compared (Bakland LK 2000).^2^ Torabinejad M, Chivian N enumerated various clinical applications of mineral trioxide aggregate. They stated that exposure of the dental pulp and periradicular tissues to microorganisms results in the development of pulpal and periradicular pathosis. To preserve vitality of pulp tissue and prevent pathological changes in the periradicular tissues, mechanical pulp exposures and carious pulp exposures in teeth with immature apexes without signs of irreversible pulpitis must be sealed. In addition, pathways of communications between the root canal system and the periodontium, such as iatrogenic perforations, must also be sealed with restorative materials that prevent bacterial leakage.^9^ Schwartz RS, Mauger M, Clement DJ, William A enumerated various uses of mineral trioxide aggregate. Mineral trioxide aggregate or MTA, is a new, biocompatible material with numerous exciting clinical applications in endodontics.^10^ Since its first description in the dental literature MTA has been used in both surgical and non-surgical applications including root-end fillings, direct pulp capping, perforation repairs in roots or furcations and apexification. It also is useful for the troublesome problems of strip perforations and perforating resorptive defects.^3^ Many materials have been used to seal the pathways of communication between root canal system and the oral cavity, as well as the periradicular tissues. The main disadvantage of these materials include microleakage, varying degrees of toxicity and sensitivity to the presence of moisture.^4^

Recently, a material called mineral trioxide aggregate (MTA) has been investigated as a potential compound to seal off the pathways of communication between root canal system and the external surface of the tooth. MTA has been used as a capping material in mechanically exposed pulps, for root end induction, repair of root perforation and as a barrier during internal bleaching of endodonti-cally treated teeth. Pulp capping and pulpectomies are indicated only in teeth with immature apexes when the dental pulps are exposed and pulp vitality should be maintained.^11^

## CONCLUSION

Mineral trioxide aggregate is a new material that possesses numerous exciting possibilities for pulpal therapy, as animal studies and clinical results are highly encouraging. No longer are immature permanent teeth with deep carious lesion or traumatic pulp exposure destined for endodontic therapy. Certainly mineral trioxide aggregate can not be used to save every tooth with pulpal involvement, however with meticulous technique it may serve as an advance pulp medicament to add to a clinician armamentarium. So the quest for an ideal material with predictable sealability, good biocompatibility and increased moisture sensitivity still exists.

## References

[1] Torabinejad M, Chivian N (1999). Clinical applications of mineral trioxide aggregate.. J Endod.

[2] Kettering JD., Torabinejad M., Cohen S, Burns RC (1999). Microbiology and immunology.. Pathways of the pulp.

[3] Islam I, Chng HK, Yap AU (2006). Comparison of the physical and mechanical properties of MTA and portland cement.. J Endod.

[4] Ribeiro DA, Sugui MM, Matsumoto MA, Duarte MA, Marques ME, Salvadori DM (2006). Genotoxicity and cytotoxicity of mineral trioxide aggregate and regular and white Portland cements on Chinese hamster ovary (CHO) cells in vitro.. Oral Surg Oral Med Oral Pathol Oral Radiol Endod.

[5] Bakland LK (2000). Management of traumatically injured pulps in immature teeth using MTA.. J Calif Dent Assoc.

[6] Camilleri J, Pitt Ford TR (2006). Mineral trioxide aggregate: a review of the constituents and biological properties of the material.. Int Endod J.

[7] Torabinejad M, Pitt Ford TR, McKendry DJ, Abedi HR, Miller DA, Kariyawasam SP (1997). Histologic assessment of mineral triox-ide aggregate as a root-end filling in monkeys.. J Endod.

[8] Trope M., Chivian N., Sigurdsson A, Cohen S, Burns RC (2002). Traumatic injuries. Pathways of the pulp.

[9] Torabinejad M, Hong CU, McDonald F, Pitt Ford TR (1995). Physical and chemical properties of a new root-end filling material.. J Endod.

[10] Schwartz RS, Mauger M, Clement DJ, William A 3rd (1999). Mineral trioxide aggregate: a new material for endodontics.. J Am Dent Assoc.

[11] Castellucci A (2003). The use of mineral trioxide aggregate in clinical and surgical endodontics.. Dent Today.

